# E-health psychological intervention in pregnant women exposed to intimate partner violence (eIPV): A protocol for a pilot randomised controlled trial

**DOI:** 10.1371/journal.pone.0282997

**Published:** 2023-03-17

**Authors:** Antonella Ludmila Zapata-Calvente, Stella Martín-de-las-Heras, Aurora Bueno Cavanillas, Karen Andreasen, Vibeke Rasch, Khalid S. Khan

**Affiliations:** 1 Brain and Behavior Research Center (CIMCYC), University of Granada, Granada, Spain; 2 Department of Forensic Medicine, University of Malaga, Málaga, Spain; 3 Instituto de Investigación Biomédica de Málaga (IBIMA), Universidad de Málaga, Málaga, Spain; 4 Department of Preventive Medicine and Public Health, University of Granada, Granada, Spain; 5 CIBER of Epidemiology and Public Health (CIBERESP), Madrid, Spain; 6 Department of Obstetrics and Gynecology, Odense University Hospital, Odense, Denmark; 7 Department of Clinical Research, University of Southern Denmark, Odense, Denmark; University of Abuja Teaching Hospital, NIGERIA

## Abstract

Intimate partner violence (IPV) during pregnancy, a condition as common as obstetrics conditions like gestational diabetes, is associated with maternal and neonatal complications. Systematic detection of IPV is not well established in antenatal screening probably because the effectiveness of protective interventions has not been evaluated. E-health interventions may be beneficial among mothers exposed to IPV. Prior to performing a full-scale effectiveness trial for such an intervention, a pilot study is required to assess the feasibility of randomising a sufficiently large number of women exposed to IPV during pregnancy. The eIPV trial is a randomised pilot study nested within a cohort of consenting mothers who screen positive for IPV in the first antenatal visit at <12 weeks’ gestation and accept an e-health package (psychological counselling by videoconference) in Spain and Denmark. Twenty eligible mothers from the above cohort will be randomised to either intervention or control. The intervention group will receive the e-health package as part of the cohort. The control group will be invited to accept a delay in the intervention (e-health package eight weeks later). After consenting to delay, the control group will provide comparative data without losing the opportunity of obtaining the intervention. We will determine estimates of rates of informed consent to randomization, and the rates of adherence and dropout following randomization. Qualitative interviews will be conducted to examine the women’s perception about the benefit of the intervention, reasons for acceptability and non-adherence, and obstacles to recruitment, randomisation and consent. The results will inform the trial feasibility and variance of key clinical outcome measures for estimation of sample size of the full-scale effectiveness trial.

## Introduction

### Background and rationale

Intimate partner violence (IPV) is one of the most common forms of violence against women and includes physical, sexual, and emotional abuse and controlling behaviour [1]. Globally, the lifetime prevalence of physical and sexual IPV for women is around 30% [2, 3]. Violence during pregnancy is more common than preeclampsia or gestational diabetes which are routinely screened for antenatally [4, 5]. In Spain, 3.6% of pregnant women have suffered physical IPV during pregnancy [6, 7]. In Denmark, 2.5% of pregnant women have reported physical violence [8]. Although the true prevalence of IPV during pregnancy in EU is unclear, it is evident that a substantial minority of women experience violence during pregnancy [9]. Moreover, pregnancy violence often continues into the postpartum period with an estimated 10% of hospitalizations due to injury in pregnancy are the result of intentional injuries inflicted upon the pregnant woman [10]. Also, IPV during pregnancy has been linked to depression, both during pregnancy and in the postpartum period [11].

To improve the health of pregnant women and their infants, it is important that targeted IPV interventions are developed and implemented as part of routine antenatal care.

E-health tools are potential solutions to deliver effective treatment and support to women exposed to IPV. eHealth interventions have the potential to provide more flexibility and safer spaces than face-to-face interventions, making it particularly useful for screening violence, empowering women and reducing exposure to IPV [12]. IPV survivors considered appropriate and necessary to be screened using eHealth strategies in antenatal care settings and perceived that video counselling is a viable and acceptable tool for pregnant women who experience IPV [13]. Trials have shown that e-health screening tools are effective in getting women to disclose or detect IPV [14–16]. Trials have also found that women who receive telephone counselling combined with other forms of support or a web-based safety decision aid kit are more likely to adopt safety behaviours than women in a control group, which were not offered e-health solutions [17–20]. It has been found that online safety planning is a feasible method among pregnant women [21].

Based on the available evidence, we have developed an e-health intervention package combining an online screening tool and video counselling sessions by trained providers for pregnant women who screen positive for IPV. Screening for IPV combined with an empowerment intervention that includes brief education and video counselling on safety planning may have potential to address repeated IPV and associated adverse health effects among pregnant women. However, prior to performing a large interventional trial, a pilot study is needed to identify barriers to recruitment, assess feasibility and acceptability of the treatment, and fine-tune study procedures.

### Objective

The objective is to assess the need and feasibility of randomising a sufficiently large number of women exposed to IPV during pregnancy in a full-scale future randomised trial. To achieve this, we will:

- estimate rates of consent to randomization, and the rates of adherence and dropout following randomization (for the use in sample size estimation)
- determine recruitment duration
- examine the women’s perception about the benefit of the intervention
- determine the reasons for acceptability, non-adherence, and obstacles to recruitment, randomisation and consent through qualitative interviews

### Trial design

A pilot randomised controlled trial (RCT), co-designed by patient input using Zelen’s design with additional qualitative evaluation, will be nested within a cohort study. To write the present protocol, the SPIRIT guidelines were followed (S1 and S2 Checklists).

**Fig 1:**
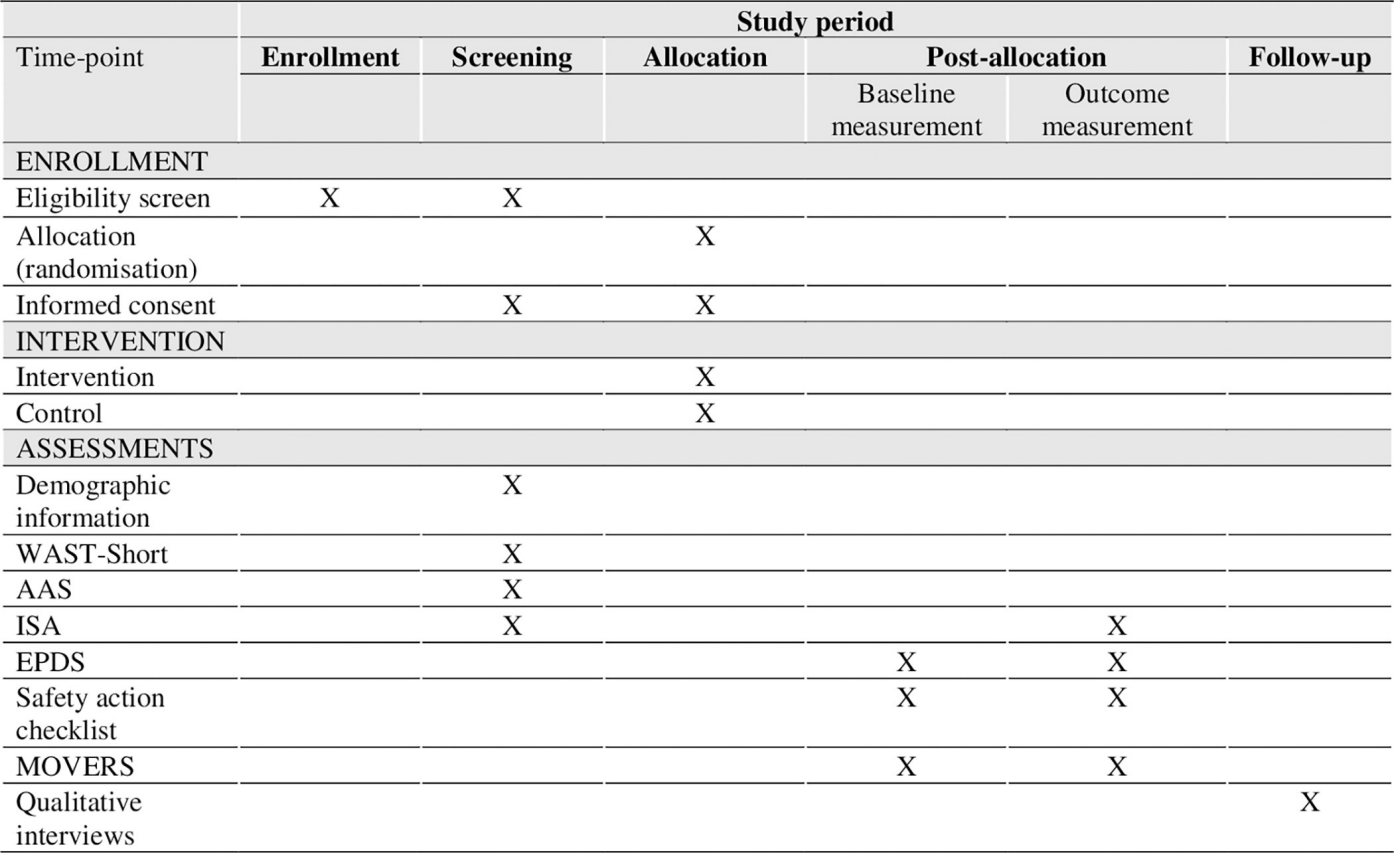
SPIRIT schedule of enrolment, interventions and assessments in eIPV trial.

**Fig 2:**
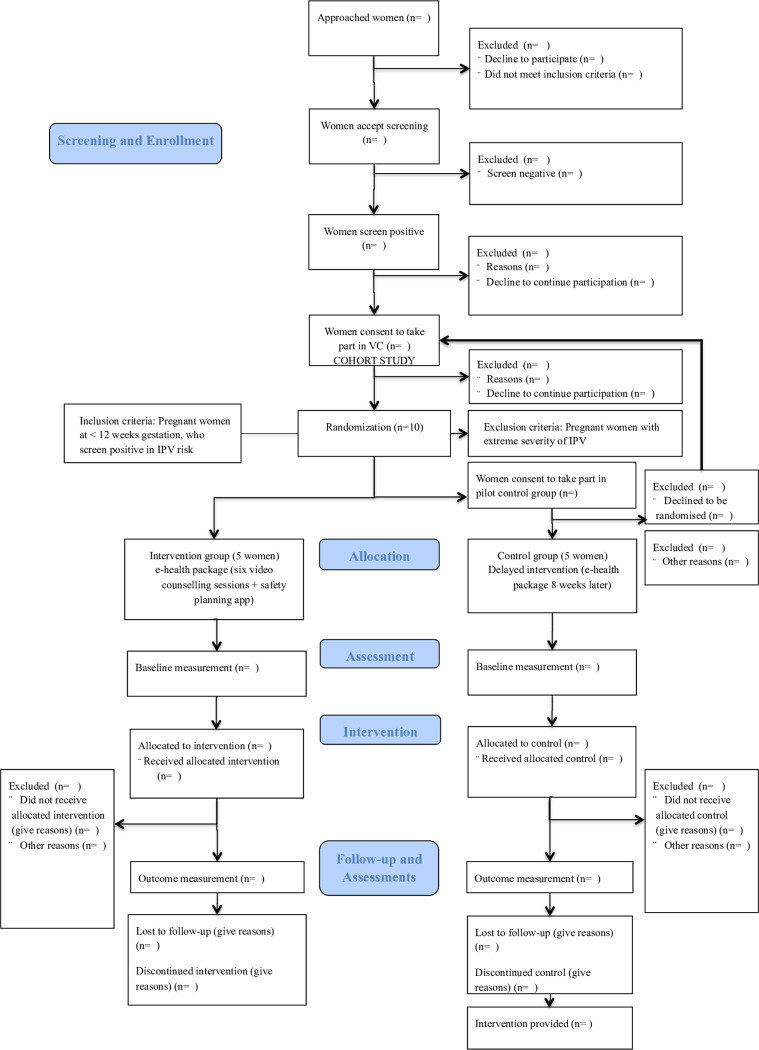
Flowchart of eIPV trial.

We will use a modified Zelen’ design with a double consent process [22–24] and a delayed intervention for the control group. In the first stage, informed consent will be sought from all pregnant women to enter a cohort study. A predetermined small number of the cohort will then be randomized, without their knowledge, to intervention or control (to see the SPIRIT schedule of enrolment, interventions and assessments in eIPV trial see Fig 1 and the Flowchart of eIPV trial in Fig 2). The intervention group will receive the e-health package as part of the cohort.

In the second stage, participants who have been assigned to the control, will be reapproached and given information about their participation in the control group. At this stage, they will be invited to accept a delay in the intervention (e-health package eight weeks later) and be asked to give the second informed consent for a delayed intervention. Those who decline will remain in the cohort study. Those in the cohort only group will not be informed about the randomization, as their subsequent follow up in the study will remain part of the cohort study to which they would already have consented in the first stage. We will substitute the women who not consent being part of the control until we reach the sample required (5 women in each country). This information will be also analysed for a future full-scale RCT.

## Methods: Participants, interventions and outcomes

### Ethics and dissemination

The trial was granted Andalusian Research Ethics Committee (S2 File; 202167133116; 202072112495; 2021128101651) and the Regional Committees on Health Research Ethics for Southern Denmark (20212000–80). Any changes to the protocol are subject to a formal amendment and may not be implemented prior to the approval by the Research Ethics Committee of each country.

### Study setting

Participants will be recruited at twenty-nine urban public primary health antenatal care centers within Andalusia (Spain) and at Odense University Hospital in the Region of Southern Denmark. Participants will be randomised to the intervention group or to the control group (Fig 2).

### Eligibility criteria

All women who fulfil the inclusion criteria will be screened and invited to receive an e-health package. Of those who accept the e-health intervention, 20 women will be randomised, 10 women in each country. In Spain and Denmark, respectively, 5 will be allocated in the intervention group and 5 will be asked to consent to be in the control group.

Inclusion criteria: pregnant women at <12 weeks gestation, who screen positive in IPV at the first antenatal visit and accept the e-health package.

Exclusion criteria: (1) women who cannot be informed about the study without their partners or other family members knowing; (2) mentally or physically incapacity to participate in the study; (3) women below 16 years in Spain or below 18 years in Denmark; (4) inability to understand Danish/Spanish, (5) lack of internet and electronic device and (6) women with extreme severity of IPV. Women selected to participate in the trial in this situation will receive an evaluation of danger before randomisation. If the severity of IPV is confirmed to be at high level of danger, these pregnant women will be routinely treated and supported according to the standard protocol in each country. Following this path, any potential risk for the women will be taken into account from the beginning of the process and women will be protected. Women who have same-sex partners will be screened, but their data will not be used for the purpose of this study.

### Recruitment: Consent procedures

In advance, a written, informed consent (in Spain the informed consent will be oral and recorded) for administering the screening of IPV, will be sought from the pregnant women at the first antenatal visit with a midwife. Those who screen positive, will also be asked for consent to receive the e-health package intervention and to complete baseline and outcome measurement questionnaires (one month after the intervention). For the pilot trial, women who consent to receive the e-health package will be randomized (following a modified Zelen’s design) into the intervention group (without their knowledge) or into the control group. Women in the control group will additionally be asked for a second informed consent, to be allocated in this group and to receive the e-health package 8 weeks later. This approach is suggested by the input of IPV survivors. A few women from the intervention and control groups of the pilot trial will be asked for consent to be contacted for a qualitative interview. Women will be informed that they have the right to refuse participation as well as to withdraw their consent at any time, without giving a reason, and that this will not affect their subsequent care. If they withdraw consent, data collected up to the point of withdrawal will be retained in the study. Participant information sheets and consent forms used for this pilot study are available on request from investigators. The informed consent provides study aims, contact details and sources for further information. Given the importance of taking into consideration the views and opinion of the participants [25], all recruitment materials have been developed with significant input from a patient and public involvement (PPI) group consisting at two participant representatives from Spain and Denmark (see the Discussion section).

### Screening procedures

Eligible women are invited by trained midwives at the first antenatal care visit to fill in an app developed for tablets for the screening of IPV during pregnancy (in Spain). In Denmark, screening for IPV is part of routine antenatal care in the Region of Southern Denmark where pregnant women are asked to fill in an electronical questionnaire on health conditions and pregnancy related life-style measures in the first trimester (PRO-data), including a screening for IPV exposure.

Women who screen positive in IPV will be invited to receive an e-health intervention (see the positivity criteria in the data collection section below).

### Randomisation procedures

The research team will consult the data server on a daily basis to identify women who screen positive in IPV and accept to receive the e-health package to randomly allocate them (using a computer-generated random numbers) in the intervention or control group through randomisation at a ratio of 1:1. IPV counsellors will be informed of the assignments to each group. Women in the intervention group will be blinded but not women in the control group.

### Study interventions and assessments

Intervention group: Women positive for IPV who accept the e-Health intervention and who have been randomly allocated in the intervention group will receive the e-health package as the rest of the cohort, as well as the baseline and outcome measurements. The e-health package will include six video counselling sessions by trained providers—a psychologist in Spain; midwives in Denmark. Women in both countries will be provided access to a mobile app for designing security plans. This will be an adapted version of the mobile app “My Plan”. To organize and host the video counselling sessions the platform “Linkello Medical” will be used in Spain and the app “My Hospital” will be used in Denmark. Both allow health providers to interact with the women in the place they prefer as long as they have Internet connection. In “Linkello Medical”, the psychologist will send an Internet link to the women by her preferred means (via mobile message or by email) and she would just have to click on the link to connect. Women will not need to register nor provide any personal data. The link will be volatile, i.e., it will be not reusable after completion of the communication. In “My Hospital”, is a software developed specifically for confidential hospital-patient communication in the Region of Southern Denmark and is already used in antenatal care as a regular communication tool. When the pregnant woman attend her first antenatal care visit, she will be introduced to My Hospital and instructed in how to access the system via the Internet or a mobile app. The midwives book the women for a video call through the system. Fifteen minutes before the video call is starting, the woman receives a notification to accept the call. Once the woman has accepted the call, the midwife can start the video consultation. This easy and confidential way of interaction will facilitate women’s participation. The mobile app for safety planning will be camouflaged to look like a common pregnancy app. The content and setup of the safety planning app was adapted from the interactive safety decision aid developed by Glass, et al. [26] which is freely available. A safety plan is a personal and practical plan, designed by a woman exposed to violence to minimize their risk of danger and exposure to violence. By digitalizing the safety plan into an app, the women will always have it available to remind them of their own strategies and resources. The safety planning element will be hidden inside the pregnancy app for security reasons; the women must log on for this element to be opened. Once a woman has logged on, the front page consists of two buttons, “Help” and “Emergency”. Both buttons consist of default contact information for relevant resources which can help a woman in case she needs help or is in an emergency situation. The woman can edit the information to fit her own needs. The app also includes the following features: “Warning signs”, where the woman can add signs that may trigger situations where violence could occur; “Strategies”‘, where the woman can note down her coping strategies; and, “Diary”, where a woman can write down anything of importance. It will also contain the feature “Knowledge about domestic violence” which will provide information and links to local and national resource’s relevant for women exposed to violence. Under another feature called “Quick Messages” a woman can enable a predetermined message to her emergency contacts. In case the woman needs to exit the app quickly, she will be able to press the “Quick-exit” button, where she will immediately return to the camouflaged part of the app. This app has been made freely downloadable to researchers and users for both android and iOS devices.

We will monitor if participants adhere to all the sessions offered for counselling and in case of non-adherence or failure to engage in counselling, the counsellor will follow specific protocol to encourage reengagement. In Spain, the psychologist will contact the woman two to three times through the preferred mean (email or phone). In Denmark, a project midwife will try to contact the woman on the preferred mean (email or phone) at 3 occasions within 6 weeks. The content of the six individually tailored sessions will be based on the Dutton’s Empowerment Model [27] and the Psychosocial Readiness Model [28]. Specifically, the contents will include (session 1) the evaluation of abusive behaviour (which will also served to protect the pregnant women is the level of violence detected is high); (session 2) safety planning, network and resources; (session 3) psycho-education (healthy relationships, cycle of violence…); (session 4) self-esteem and self-care and (sessions 5 & 6) empowerment, choice making and problem solving. The video consultations will be planned for every 2 weeks at a time where the women feel comfortable. However, schedule flexibility will always prevail to promote women participation. Criteria for discontinuing or modifying allocated interventions will be applied to women with an elevated risk for IPV who will be routinely treated according to the standard protocol in each country. In other words, if during the interventions the health providers detect an increased on the level of violence, they will apply the established national protocols to protect the pregnant women.

Control group: IPV positive women who accept the e-Health intervention package will be asked for a second consent to receive a delayed intervention (8 weeks later) and to complete the baseline and outcome measurements. Women can request to leave the control group at any time and receive the intervention immediately (in which case the data will be part of the cohort study).

Assessments and data collection: data on socio-demographic characteristics and partner violence will be collected during the screening process. The validated screening questionnaires to detect IPV will be the short form of the Women Abuse Screening tool (WAST-Short) [29], a 2-items tool that measures conflict and tension and the Abuse Assessment Screen (AAS) [30], a 5-item screening questionnaire including emotional, physical, and sexual violence as well as fear of partner within the past year or ever. Data from previous studies support the reliability and validity of the WAST [31] and the AAS [30]. In Spain, a woman is initially screened positive for exposure to IPV in the AAS if she answers “yes” to one or more of the five questions if the perpetrator is the partner or ex-partner. In Denmark, a woman screen positive if she answers “yes” to having been afraid of their partner or ex-partner or to having been exposed to emotional, physical or sexual violence within the past 12 months by a partner or ex-partner in the AAS. In Spain, a woman screens positive in WAST-Short if she meets one of the two original criteria: (1) having some or a lot of tension and/or some or a lot of difficulties to solve problems with her partner (all positive responses receive 1, the cut-off = 2) or (2) if she indicates having a lot of tension and/or a lot of difficulties (extreme responses receive 1, cut-off = 1). In Denmark, woman screens positive in WAST-Short if she meets the second criteria. If the women initially are screened positive, the screening questionnaires will automatically be followed by the Index of Spouse Abuse (ISA) questionnaire [29]. The ISA questionnaire is a detailed 30-item questionnaire about IPV (emotional, physical and sexual) in order to confirm the IPV and evaluate the severity of the violence. Two different scores are computed: ISA-P (physical abuse) and ISA-NP (non-physical abuse). The scores range from 0 to 100. In Denmark, the cut-off scores of 10 for ISA-P, and 25 for ISA-NP developed by Hudson and McIntosh will be used [32]. In Spain, based on the Spanish validation of the ISA tool [33] that gives different weights to the original items, cut-off scores of 6 for ISA-P, and 14 for ISA-NP will be applied. The reliability (α) of the ISA range from .82 [33] to .96 [32].

Quantitative questionnaire data will be collected before and after the e-health intervention. Exposure to IPV will be assessed by use of the ISA tool, and post-natal depression will be assessed by use of The Edinburgh Postnatal Depression Scale (EPDS, α = .81) which is a 10-item validated questionnaire designed to detect postnatal depression [34]. Further, participants will be asked to assess their ability to carry out safety behaviour actions by use of a revised version of the 22-item safety action checklist (α = .75) [35] and to complete a Measure of Victim Empowerment Related to Safety (MOVERS; α = .74-.88) [36] All the data will be entered into a bespoke secure online study database using a unique study ID for each participant. The health providers will check the items of the ISA related to physical IPV and the psychological IPV items measuring fear of the partner to determine if the women invited to the pilot are at extreme level of IPV risk. In those women who respond positive to the above items, before randomisation the providers will evaluate the level of danger with the Severe Intimate Partner Violence Risk Prediction Scale-Revised (EPV-R, α = .72) [37]. If the level of danger is confirmed to be high, women will not be included in the study and will be routinely treated and supported according to the standard protocol in each country.

Qualitative data will be collected through individual in-depth interviews with a few women at any point of the flow diagram (Fig 2) to explore their opinion and experiences of the study procedures and intervention. Additionally, we will conduct interviews with the IPV counsellors (midwives in Denmark and psychologist and midwives in Spain) to explore their experiences with the delivery of the intervention and their opinion about a future full-randomised controlled trial. A semi-structured interview guide will be developed for the interviews using the Model for Assessment of Telemedicine Applications (MAST) as theoretical framework [38] with focus primarily on the following domains: users’ perspectives, their safety, ethical and organisational aspects; health problems and technology. All participants will give written consent prior to the interviews starting and data collection will stop once data is saturated. The interviews will be conducted in Danish/Spanish and audio recorded, and will be transcribed and analysed by use of a thematic content analysis using a combined inductive-deductive approach [39]. A coding frame will be developed, and themes will deductively be derived from MAST and supplemented with categories that inductively arise from the data. See Fig 1 for an overview of intervention and assessments.

Data will be collected by trained health personal and researchers in the STOP (Stop Intimate Partner Violence in Pregnancy, https://stop-ipv.eu) Project. On reasonable request, protocol, data collection forms and plans for statistical analysis will be available from investigators. In case of failure to provide data in the follow-up assessments, we will follow the above described STOP protocol for follow up. In Spain, the participant will be contacted by the preferred mean: phone or email. If a woman indicates she wants to be contacted by phone, the psychologist will make a maximum of three phone calls at different times and days. If the contact is made by email or phone message, the counsellor will send a maximum of two attempts leaving between both at least three days. In Denmark, women in the project will indicate if they prefer to be contacted by phone or email at the inclusion. If an included woman is unreachable, a project midwife will try to establish contact at 3 different occasions within 6 weeks. Rate of failure to obtain data will be one of the outcome measures.

Relevant concomitant care and interventions that are permitted during the trial: Women in both the intervention and control groups would be able to benefit from the standard care available for women exposed to IPV thorough the usual provision made by social and health services at any time during the study, if necessary. Indeed, one of the video-counselling sessions will be dedicated to inform women about the available resources in their community. IPV counsellors will specify if women in the intervention and the control group are in need of these resources during the time of the pilot as a valuable information for the future full-scale trial.

To accomplish the objectives, we will collect the following information:

Primary outcome:

- Number of IPV positive women, who consented to receive e-health package and consented to randomization in the control group computed as a rate.

Secondary outcomes:

- Number of women who consented to receive e-health package computed as a rate.
- Number of women in the intervention group where complete outcome data were obtained computed as a rate.
- Number of women in the control group where complete outcome data were obtained computed as a rate.
- Number of women where there was a study protocol violation computed as a rate.
- Recruitment duration (in days) to get the pilot sample (5 women for the intervention group and 5 women for the control group, in each country).
- Benefit of the intervention perceived by women through the information obtained in qualitative interviews.
- Perception about the duration of delay (the duration in the future full-scale trial will be determine according to the observations derived of the pilot study and the information provided in the qualitative interviews).
- Reasons for acceptability, non-adherence, and obstacles to recruitment, randomisation, consent and follow-up (through qualitative interviews).

Participant timeline

The participant timeline is illustrated in Fig 1.

### Statistics

#### Sample size calculation

As this is a feasibility pilot study, power calculation for sample size determination is not an obligatory requirement [40]. The pilot study will not be powered to detect statistical differences in key clinical outcomes. We will review patient and staff feedback before finalising a protocol for a full-scale multicentre RCT.

#### Statistical analysis

To determine the feasibility, we will perform the analysis against established stop-go rules according to the CONSORT statement for pilot and feasibility trials [41]. The feasibility analysis will focus on the outcomes described above. For example, we will compute the percentage and 95% confidence intervals (CI) of IPV positive women, who consented to receive e-health package and consented to randomization in the control group, following the same analytical approach than other pilot studies [42].

#### Progression criteria

Table 1 outlines the main criteria that will be considered to assess the feasibility of a full-scale RCT. In addition, our qualitative findings will also be used to support the decision-making around progression to a full-scale trial. For example, if a progression criterion outlined in Table 1 does not meet the threshold for progression, but we have developed a qualitative understanding of why this occurred and how it could be improved, then it may still be possible to proceed with the full trial. The research team of the STOP project will have access to these interim results.

**Table 1:** Suggested progression criteria in eIPV trial.

Feasibility objectives and related data to be collected	Go criteria to proceed to full trial	Criteria to reassess and adjust full trial protocol	Stop criteria
Study population			
1. Consent rate of eligible women	Rate >25% of eligible women agreeing to participate.	Rate between 11% and 24% women agreeing to participate	Rate <10% of eligible women agreeing to participate
Study outcomes			
2. Proportion of women in either intervention or control group for whom the allocated treatment is adhered to.	Adherence to allocated treatment in >80% of study sample.	Adherence to allocated treatment in between 51% and 79% of study sample.	Adherence to allocated treatment in <50% of study sample.
RCT process			
3. Collection of data on clinical outcomes	Complete data available of >80% of study sample.	Missing data between 21% and 49% of study sample.	Data missing of >50% of study sample.

## Trial management and monitoring

### Data management

All data management will be undertaken by the University of Granada (UGR) (Spain) and Odense University Hospital (Denmark). In Spain, standard operating procedures will be in place for the collection and handling of data. All study data will be entered directly by trained and delegated research staff included in the STOP project into a secure, bespoke electronic trial database with inbuilt range checks set up and hosted by the University of Granada. User accounts will be allocated and managed centrally by the trial coordinator and restricted to appropriate site level access. Data collected on the forms and entered into the electronic database will only identify the participants by a unique trial number. No identifiable data will be stored in the trial database.

In Denmark, study data are collected by trained project midwives from the STOP project following a manual for data collection and will be entered directly to a secure web-based database designed to support data capture for research studies with inbuilt range checks (REDCap) (project-redcap.org). User access to the database is restricted and assigned by the Danish trial coordinator. Data is entered into the database with a unique study trial number, and there will be no identifiable data in the database. Data with invalid study trial numbers, values outside the range or follow-up id that do not match the baseline study trial number, will be excluded.

### Trial management

The trial is managed and run by the University of Granada and Odense University Hospital. Both centers are responsible for safety reporting, coordination of trial committees, statistical analysis and reporting, trial monitoring, database management and case report form design.

### Pilot trial oversight

An advisory board has been established to oversee and monitor the trial conduct and patient safety. It is chaired by an independent professor from Norway (Dr Mirjam Lukasse), with two other independent IPV experts from Spain and Denmark (Prof Tine Gammeltoft and Dr Carmen Vives Cases). The advisory board provides overall supervision of the trial and ensures that it is being conducted according to the protocol, good clinical practice and relevant regulations. The advisory board also monitor trial progress in relation to recruitment, data capture and completeness, protocol adherence and deviations and subject withdrawals. The board will meet at the request of the investigators. It will be responsible for reviewing the trial data and assessing whether there are any safety issues that need to be brought to the attention of the sponsor, or any ethical reasons why the trial should not continue. Given the low risk of the study intervention and that it is non-blinded, no separate data safety monitoring committee will be established. The sponsor retains the right to audit the study, including any study site or central facility.

### Protecting participants and safety reporting

By taking part in the study, participants will automatically enter a project where a greater level of care will become available to them. The study has well-established security protocols. The screening questionnaires will capture the severity of violence permitting us to refer women at high risk of danger to the relevant national authorities responsible their protection. During of the first videocounselling session, even if the questionnaires did not register a high level of risk, the danger to the life of the participants will be specifically evaluated. If a risk of danger is detected, the same protocol as above will be applied. All the videocounselling sessions will be carried out with a high level of vigilance concerning danger. It should be noted that the intervention itself (as explain above) contains features that are protective, for example, a link that expiries after the counseling sessions, a protection plan back by easy-to-use features to activate emergency contacts, camouflage of the app and emergency exit button. The videocounselling providers will receive in-session protection training, for example, to change the topic of the conversation in case the partner shows up unexpectedly during the sessions. Videoconsellors and women will talk about these options at the beginning of the intervention; in this way both will know what to do if something like this happens.

For safety reporting, if severe or life-threatening abuse of a pregnant woman is identified by the IPV counsellors, the principal investigator will be notified and the woman will be treated according to the standard protocol in each country. The study does not add any risk or harm for the women, we will therefore not provide a post-trial care for compensation. On the contrary, it is anticipated that video counselling addressing safety behavior and safety planning has the potential to increase safe behaviors and thus decrease IPV exposure. We also anticipate that the participating midwives will experience a greater competence and confidence in approaching the topic of violence and handling women who are exposed to IPV. Women will benefit from health professionals who enquire in an appropriate way about violence. In addition, information will be provided about IPV community resources which the women may make use of after the e-health intervention is finished.

#### Patient and public involvement

Two women previously exposed to IPV (one Spanish and one Danish) form the target group representatives. They will help develop a more pregnant women-centred information sheet in line with the well-known need to involve citizens in science [43]. The qualitative research embedded within this pilot study will prove integral in evaluating how the consent materials and processes were received in practice.

## Discussion

Even though numerous intervention models to address IPV have been developed, current efforts suffer from limitations. First, IPV services are often not integrated within routine health service delivery, and second, models for integrating IPV service in health service delivery where developed tend not to have generalizability to the European context [14–20]. To address the personally and politically sensitive problems associated with IPV among pregnant women, effective, sustainable, and culturally appropriate health system-based interventions need development. If found effective, e-health interventions would be suitable for incorporation in these care pathways.

Screening for IPV combined with an empowerment intervention that includes education and video counselling on safety planning may have potential to address repeated IPV and associated adverse health effect among pregnant women. The option of receiving counselling and support through video consultation during antenatal care presents an opportunity for more accessible and flexible care addressing some of the barriers associated with in-person care, such as travel distance and time, travel costs, and the stigma of seeking help. In addition, patients may be more motivated to seek and continue treatment if they are in a familiar environment of their own choice and can avoid stressful situations, such as navigating a hospital or maternity facility. The development of the video counselling intervention will capture the need for safety and adhere to strict security features preventing the risk of women’s partners prying on their online activity. These provisos need underpinning effectiveness evidence.

Prior to performing a large interventional trial, a pilot study is needed to identify barriers to recruitment, assess feasibility and acceptability of the treatment, and fine-tune study procedures. No RCT has previously assessed an e-health intervention in IPV among pregnant women in comparison with a control group (with a delay intervention). In this pilot trial, we chose to perform randomisation with a modified Zelen’s design rather than simple randomisation for several reasons. Participants who take part in standard RCTs will make a judgment of their preferred treatment and often expect to be allocated to the treatment group [44]. If this does not occur, it can be followed by dissatisfaction and distrust in those who approached them to take part [45]. Consequently, randomization to a control group may lead to dropout after allocation. The original Zelen design involves randomization before consent, with consent only required from those allocated to the intervention, whereas the control group receive their usual care [24]. Baseline data and outcomes are collected from medical records (with ethical approval). However, it is not possible to have interaction with the control group during follow-up, as they are not informed of their presence in a study. Taking all of this into consideration, we hypothesized that women will accept the intervention because they perceive that it is needed for their support and protection. If they perceive it like this, they may not want to be randomized into control group or they may drop out after been randomized to usual care instead of intervention. To overcome this difficulty with acceptance of allocation to the control group, we followed the input of IPV survivors in a focus group we conducted [13]. Amongst other things the survivors informed us that they would prefer delayed intervention instead of usual care in a control group. The opinion of the participant representative in our project affirmed their support for this approach. A systematic review concluded that a delayed intervention could be an effective way of minimizing dropout [46]. In our pilot, thus we chose to offer to women of the control group a delay in the intervention. It is this specific challenge that is addressed in our pilot study. We wish to estimate the rates of acceptance of randomization primarily. We do not aim to estimate effect sizes. The approaches recommended for use of standardized effect sizes for pilot study sample sizes estimation are not applicable [47]. Our analytic approach will compute simple statistic with 95% CI and we will employ qualitative judgment in assessing the need to prepare a full-scale RCT.

Beyond the information described to be collected during this pilot, we will capture other relevant information in the cohort study useful for planning the future full-scale randomised control trial: number of women who were approached and agreed to fill in the IPV screening question, number of women where there was a study protocol violation, number of women exposed to physical IPV captured by the screening questions, number of pregnant women exposed to physical IPV that accept to participate in the intervention and in the control group, number of women willing to participate in follow-up interviews, the acceptability of the e-health package for ongoing IPV prevention and the acceptability of video-counselling for IPV prevention in terms of compliance [48] to the scheduled counselling sessions.

In conclusion, the pilot study nested within the cohort study will allow us to obtain information about the rates of IPV in pregnancy, the acceptability of an e-health intervention and the availability of participants for randomisation into an effectiveness trial. These results will inform us about the feasibility and variance of key clinical outcome measures for estimation sample size of the full-scale effectiveness trial.

### Trial status

Trial registration number: NCT04978064 (S1 File). Protocol version 1.0, 20 April 2021. The proposed start date of randomised participant recruitment: September, 2021. The proposed project recruitment completion date of any participant including those not randomised: September, 2022. The proposed end of follow-up of all participants including those randomised: September, 2022.

### Confidentiality

In order to protect confidentiality before, during, and after the trial, personal information about potential and enrolled participants will be collected and saved during the screening process in a secured data base and it will be only accessed by authorised research investigators of the STOP project. The information of the baseline and outcome measurements will be saved in different databases, and both could be only aggregated by the research members by the birthdate and telephone number of the women.

### Declarations of interests

Financial and other competing interests for principal investigators for the overall trial and each study site.

### Access to data

Research team members from the STOP project will have access to the final pilot trial dataset only for research purposes according to the objectives established in this protocol. We will share the anonymized dataset and output of the statistical analysis openly and transparently on completion of the study providing it in a repository or a supplementary file at the time of submission of the manuscript containing results of the completed study.

### Dissemination policy

We will share the eHealth package with relevant stakeholders in order to upscale it across the EU as well as globally to better combat IPV. Concrete dissemination activities include the production of research papers, guidelines and white papers detailing the eHealth intervention, which will be made available on the STOP project website and presented at relevant professional conferences reaching both health care professionals and other relevant stakeholders and policymakers.

### Consent for publication

All relevant data from this study will be submitted to peer-reviewed journals for publication following the completion of the study in line with sponsor publication policy. Data will be captured for all study participants, and no patient identifiable data will be used in any publications. The sponsor retains the right to review all publications prior to submission or publication. Responsibility for ensuring accuracy of any publication from this study is delegated to the chief investigator. Authorship will be assigned in compliance with International Committee of Medical Journal Editors (ICMJE) guidelines.

The Advisory board is comprised by Dr. Mirjam Lukasse (Norway), Dr. Tine Gammeltoft (Denmark), Dr. Carmen Vives Cases (Spain) and two participant representatives from Denmark and Spain whom prefer to remain anonymous.

## Supporting information

S1 Checklist SPIRIT 2013 checklist. (DOC)

S2 Checklist All items from WHO trial registration data set. (DOCX)

S1 File eIPV clinical trial registry. (PDF)

S2 File Protocol approved by ethics committee. (PDF)
