# Supplementary material for: E-health psychological intervention in pregnant women exposed to intimate partner violence (eIPV): A protocol for a pilot randomised controlled trial
Source: PLoS One. 2023 Mar 17;18(3):e0282997. doi: 10.1371/journal.pone.0282997 (PMC10022801; doi:10.1371/journal.pone.0282997)
Supplement: S2 Checklist — (DOCX) [file pone.0282997.s002.docx]

WHO Trial Registration Data Set (Version 1.3.1) <https://www.who.int/clinical-trials-registry-platform/network/who-data-set>

2b All items from the World Health Organization Trial Registration Data Set

Example of hoe to be completed: <http://www.spirit-statement.org/data-set/>

| **Data category** | **Information** |
| --- | --- |
| 1. Primary Registry and Trial Identifying Number | ClinicalTrials.gov  ID number: NCT04978064 |
| 1. Date of Registration in Primary Registry | 27/07/2021 |
| 1. Secondary Identifying Numbers | - Andalusian Research Ethics Committee, 202167133116 - Regional Committees Health Research Ethics Southern Denmark, 20212000-80 |
| 1. Source(s) of Monetary or Material Support | Financial support from Justice Programme (JUST) Rights, Equality and Citizenship Programme (REC) REC-RDAP-GBV-AG-2019  The European Commission have reviewed the funding application, and will have oversight of study progress, but neither of the funders have any role in the study design, data collection and analysis, decision to publish, or preparation of the manuscript. |
| 1. Primary Sponsor | University of Granada |
| 1. Secondary Sponsor(s) | University of Southern Denmark  Odense University Hospital |
| 1. Contact for Public Queries | Email address, telephone number and postal address of the contact who will respond to general queries, including information about current recruitment status.  [antonellalzc@ugr.es](mailto:antonellalzc@ugr.es)  Telephone number: 958246270 (Department of Social Psychology. University of Granada)  Postal address: Campus Universitario de Cartuja, Granada (18011), Spain |
| 1. Contact for Scientific Queries | Principal investigator Dr. Antonella Ludmila Zapata-Calvente  Email: [antonellalzc@ugr.es](mailto:antonellalzc@ugr.es)  Telephone number: 958246270 (Department of Social Psychology. University of Granada)  Postal address: Campus Universitario de Cartuja, Granada (18011), Spain  Affiliation: Brain and Behavior Research Center (CIMCYC) and Faculty of Psychology; University of Granada, Spain. |
| 1. Public Title | E-health Psychological Intervention in Pregnant Women Exposed to Intimate Partner Violence (eIPV) |
| 1. Scientific Title | E-health Psychological Intervention in Pregnant Women Exposed to Intimate Partner Violence (eIPV): a Pilot Randomized Controlled Trial |
| 1. Countries of Recruitment | Spain  Denmark |
| 1. Health Condition(s) or Problem(s) Studied | Intimate Partner Violence in Pregnant Women |
| 1. Intervention(s) | **Intervention group: Behavioral e-health psychological counselling:**  Women positive for IPV who accept the e-Health intervention and who have been randomly allocated in the intervention group will receive the e-health package as the rest of the cohort, as well as the baseline and outcome measurements. The e-health package will include six video counselling sessions by trained providers and the access to a mobile application for designing security plans, an adapted version of the mobile application "My Plan". The content of the six individually tailored sessions will be based on the Dutton's Empowerment Model and the Psychosocial Readiness Model.  **Control group: Usual care (delayed behavioral e-health psychological counselling)**  Control group: women positive in IPV who accept the e-Health intervention package will be asked for a second consent to receive a delayed intervention (8 weeks later) and to complete as the baseline and outcome measurements. Women can request to leave the control group at any time and to receive the intervention immediately (in which case they data will be part of the cohort study). The e-health package will include six video counselling sessions by trained providers and the access to a mobile application for designing security plans, an adapted version of the mobile application "My Plan". The content of the six individually tailored sessions will be based on the Dutton's Empowerment Model and the Psychosocial Readiness Model. |
| 1. Key Inclusion and Exclusion Criteria | Minimum Age: 16  Sex: Female  Inclusion Criteria:   - Pregnant women at <12 weeks gestation, who screen positive in IPV at the first antenatal visit and accept the e-health package.   Exclusion Criteria:   - Women who cannot be informed about the study without their partners or other family members knowing - Women mentally or physically incapacity to participate in the study - Women below 16 years in Spain or below 18 years in Denmark - Inability to understand Danish/Spanish - Lack of internet and electronic device - Women with extreme severity of IPV. Women selected to participate in the trial in this situation will receive a danger assessment before randomisation and if the severity of IPV is confirmed, they will be routinely treated and supported according to the standard protocol in each country. Women who have same-sex partners will be screened, but their data will not be used for the purpose of this study. |
| 1. Study Type | Study type consists of:  Interventional study  Method of allocation: randomized  Masking: Single (Participant).Women in the intervention group will be blinded but not women in the control group.  Assignment: parallel  Purpose: supportive care  A pilot randomised controlled trial (RCT), co-designed by patient input using a modified Zelen’s design with additional qualitative evaluation, will be nested within a cohort study. |
| 1. Date of First Enrollment | The proposed start date of randomised participant recruitment: September, 2021. The proposed project recruitment completion date of any participant including those not randomised: September, 2022. The proposed end of follow-up of all participants including those randomised: September, 2022. |
| 1. Sample Size | Sample Size consists of: 20 (10 pregnant women in each country)  Number of participants that the trial plans to enrol in total: : 20 (10 pregnant women in each country) |
| 1. Recruitment Status | Recruitment Status: Pending: participants are not yet being recruited or enrolled at any site |
| 1. Primary Outcome(s) | Primary Outcome Measure:  1. Consent rate for a future full-scale RCT trial Rate of women who were positive in IPV, consent to receive e-health package and consent to randomization in the control group.  [Time Frame: Three to nine months] |
| 1. Key Secondary Outcomes | Secondary Outcome Measure:  2. Positivity rate of the cohort study (useful for planning the future full-scale randomised control trial): Rate of women who were positive in IPV and consent to receive e-health package  [Time Frame: Three to nine months]  3. Completion rate in the intervention group a future full-scale RCT trial Rate of women who were recruited to intervention group, and for whom complete outcomes were obtained.  [Time Frame: Three to nine months]  4. Completion rate in the control group a future full-scale RCT trial Rate of women who were recruited to control group, and for whom complete outcomes were obtained.  [Time Frame: Three to nine months]  5. Recruitment duration for a future full-scale RCT trial Recruitment duration (in days) to get the pilot sample (5 women for the intervention group and 5 women for the control group, in each country)  [Time Frame: Three to nine months]  6. Benefit of the intervention a future full-scale RCT trial Perception of the intervention by women participating in the pilot through the information obtained in qualitative interviews.  [Time Frame: Three to nine months]  7. Perception about the delay of the intervention of the control group for a future full-scale RCT trial Perception about the duration of delay of the intervention of women in the control group (provided in the qualitative interviews).  [Time Frame: Three to nine months]  8. Reasons for acceptability, non-adherence, and obstacles for a future full-scale RCT trial Reasons for acceptability, non-adherence, and obstacles to recruitment, randomization, consent and follow-up (provided in the qualitative interviews).  [Time Frame: Three to nine months]  9. Follow up rate for a future full-scale RCT trial Rate of failure to obtain data in the follow-up.  [Time Frame: Three to nine months] |
| 1. Ethics Review | The ethics review process information of the trial record in the primary register database. It consists of:  Andalusian Research Ethics Committee  Status: Approved (202167133116)  Date of approval: 07/06/2021  Name and contact details of Ethics committee(s):  Demetrio Mariano Aguayo Canela  E-mail: [cceiba.csalud@juntadeandalucia.es](mailto:cceiba.csalud@juntadeandalucia.es)  Consejería de Salud y Familias. Avda. de la Innovación s/n. Edificio Arena 1, 41071, Sevilla. Spain.  Regional Committees Health Research Ethics Southern Denmark  Status: Approved (20212000-80)  Date of approval: 10/05/2021  Name and contact details of Ethics committee(s):  Christina Sølvsten Fly  *Administrativ koordinator*  *Kvalitet og Forskning*  *De Videnskabsetiske Komitéer for Region Syddanmark*   \| E-mail: \| [Christina.Soelvsten.Fly@rsyd.dk](mailto:Christina.Soelvsten.Fly@rsyd.dk) \| \| --- \| --- \| |
| 1. Completion date | The proposed project recruitment completion date of any participant including those not randomised: September, 2022 |
| 1. Summary Results | Date of posting of results summaries: after follow-up  Adverse events: None  URL link to protocol file(s) with version and date:  <https://clinicaltrials.gov/ct2/show/NCT04978064?term>=  NCT04978064&draw=2&rank=1 First posted July 27, 2021 |
| 1. IPD sharing statement | We will collaborate in an approved, registered Individual Participant Data (IPD) metaanalysis  Supporting Information:-   - Study Protocol - Informed Consent Form (ICF) - Clinical Study Report (CSR)   Time Frame:   - Starting 6 months after publication   Access Criteria:  On reasonable request, protocol, data collection forms and published results are available from investigators. Sharing would need to be comply with data protection laws. |
