# Supplementary material for: E-health psychological intervention in pregnant women exposed to intimate partner violence (eIPV): A protocol for a pilot randomised controlled trial
Source: PLoS One. 2023 Mar 17;18(3):e0282997. doi: 10.1371/journal.pone.0282997 (PMC10022801; doi:10.1371/journal.pone.0282997)
Supplement: S1 File — (PDF) [file pone.0282997.s003.pdf]

ClinicalTrials.gov Protocol Registration and Results System (PRS) Receipt
Release Date: July 15, 2021

ClinicalTrials.gov ID: NCT04978064

Study Identification

Unique Protocol ID: eIPV

Brief Title: E-health Psychological Intervention in Pregnant Women Exposed to Intimate Partner Violence (eIPV)

Official Title: E-health Psychological Intervention in Pregnant Women Exposed to Intimate Partner Violence (eIPV): a Pilot Randomised Controlled Trial

Secondary IDs: 202167133116 [Andalusian Research Ethics Committee]
20212000-80 [Regional Committees Health Research Ethics Southern Denmark]

Study Status

Record Verification: July 2021

Overall Status: Not yet recruiting

Study Start: September 1, 2021 [Anticipated]

Primary Completion: September 1, 2022 [Anticipated]

Study Completion: September 1, 2022 [Anticipated]

Sponsor/Collaborators

Sponsor: Universidad de Granada

Responsible Party: Principal Investigator
Investigator: Antonella Ludmila Zapata Calvente [azapatacalvente]
Official Title: Principal Investigator
Affiliation: Universidad de Granada

Collaborators: University of Southern Denmark
Odense University Hospital

Oversight

U.S. FDA-regulated Drug: No

U.S. FDA-regulated Device: No

U.S. FDA IND/IDE: No

Human Subjects Review: Board Status: Approved
Approval Number: 202167133116
Board Name: Comité de Ética de la Investigación (CEIM/CEI) Provincial de Granada

Board Affiliation: Red de Comités de Ética del Sistema Sanitario Público de Andalucía
Phone: 0034 671593204
Email: ceigranada.hsc.sspa@juntadeandalucia.es
Address:

Junta de Andalucía, Granada, Spain

Data Monitoring: Yes

FDA Regulated Intervention: No

Study Description

Brief Summary: The investigators will assess the need and feasibility of randomising a sufficiently large number of women exposed to IPV during pregnancy in a full-scale future randomised trial. To achieve this, the investigators will:

a. estimate rates of consent to randomization, and the rates of adherence and dropout following randomization (for the use in sample size estimation)
b. determine recruitment duration
c. examine the women's perception about the benefit of the intervention
d. determine the reasons for acceptability, non-adherence, and obstacles to recruitment, randomisation and consent through qualitative interviews

Detailed Description: Introduction. Intimate partner violence (IPV) during pregnancy, a condition as common as obstetrics conditions like gestational diabetes, is associated with maternal and neonatal complications. Systematic detection of IPV is not well established in antenatal screening probably because the effectiveness of protective interventions has not been evaluated. Among mothers exposed to IPV, e-health interventions during pregnancy may be beneficial. Prior to performing a full-scale effectiveness trial for such an intervention, a pilot study is required to assess the need and feasibility of randomising a sufficiently large number of women at exposed to IPV during pregnancy.

Methods. The eIPV trial is a randomised pilot study nested within a cohort of consenting mothers at <12 weeks' gestation who screen positive for IPV in the first antenatal visit and accept an e-health package (psychological counselling by videoconference) in Spain and Denmark. Twenty eligible mothers from the above cohort will be randomised to either intervention or control. The intervention group will receive the e-health package as part of the cohort. The control group will be invited to accept a delay in the intervention (e-health package eight weeks later). After consenting to delay, the control group will provide comparative data without losing the opportunity of obtaining the intervention. The investigators will determine estimates of rates of informed consent to randomization, and the rates of adherence and dropout following randomization. Qualitative interviews will be conducted to examine the women's perception about the benefit of the intervention, reasons for acceptability and non-adherence, and obstacles to recruitment, randomisation and consent. The results will inform the feasibility and variance of key clinical outcome measures for estimation of sample size of the full-scale effectiveness trial.

Comment. The pilot study nested within the cohort study will allow us to obtain information about the rates of IPV in pregnancy, the acceptability of an e-health intervention and the availability of participants for randomisation into a large effectiveness trial.

Conditions

Conditions: Pregnancy Related
Violence, Gender-Based
Intimate Partner Violence

Keywords: pilot randomized controlled trial
pregnancy
intimate partner violence
e-health intervention
counselling

Study Design

Study Type: Interventional

Primary Purpose: Supportive Care

Study Phase: N/A

Interventional Study Model: Parallel Assignment
A pilot randomised controlled trial (RCT), co-designed by patient input using a modified Zelen's design with additional qualitative evaluation, will be nested within a cohort study.

Number of Arms: 2

Masking: Single (Participant)
Women in the intervention group will be blinded but not women in the control group.

Allocation: Randomized

Enrollment: 20 [Anticipated]

Arms and Interventions

Arms	Assigned Interventions
Experimental: Intervention group<br>Behavioral e-health psychological counselling	Behavioral: e-health psychological counselling<br>Intervention group: Women positive for IPV who accept the e-Health intervention and who have been randomly allocated in the intervention group will receive the e-health package as the rest of the cohort, as well as the baseline and outcome measurements. The e-health package will include six video counselling sessions by trained providers and the access to a mobile application for designing security plans, an adapted version of the mobile application "My Plan". The content of the six individually tailored sessions will be based on the Dutton's Empowerment Model and the Psychosocial Readiness Model. Control group: women positive in IPV who accept the e-Health intervention package will be asked for a second consent to receive a delayed intervention (8 weeks later) and to complete as the baseline and outcome measurements. Women can request to leave the control group at any time and to receive the intervention immediately (in which case they data will be part of the cohort study).
Control group	Behavioral: e-health psychological counselling

Arms	Assigned Interventions
Usual care (delayed behavioral e-health psychological counselling)	Intervention group: Women positive for IPV who accept the e-Health intervention and who have been randomly allocated in the intervention group will receive the e-health package as the rest of the cohort, as well as the baseline and outcome measurements. The e-health package will include six video counselling sessions by trained providers and the access to a mobile application for designing security plans, an adapted version of the mobile application "My Plan". The content of the six individually tailored sessions will be based on the Dutton's Empowerment Model and the Psychosocial Readiness Model. Control group: women positive in IPV who accept the e-Health intervention package will be asked for a second consent to receive a delayed intervention (8 weeks later) and to complete as the baseline and outcome measurements. Women can request to leave the control group at any time and to receive the intervention immediately (in which case they data will be part of the cohort study).

Outcome Measures

Primary Outcome Measure:

1. Consent rate for a future full-scale RCT trial
   Rate of women who were positive in IPV, consent to receive e-health package and consent to randomization in the control group.

   [Time Frame: Three to nine months]

Secondary Outcome Measure:

2. Positivity rate of the cohort study (useful for planning the future full-scale randomised control trial):
   Rate of women who were positive in IPV and consent to receive e-health package

   [Time Frame: Three to nine months]
3. Completion rate in the intervention group a future full-scale RCT trial
   Rate of women who were recruited to intervention group, and for whom complete outcomes were obtained.

   [Time Frame: Three to nine months]
4. Completion rate in the control group a future full-scale RCT trial
   Rate of women who were recruited to control group, and for whom complete outcomes were obtained.

   [Time Frame: Three to nine months]
5. Recruitment duration for a future full-scale RCT trial
   Recruitment duration (in days) to get the pilot sample (5 women for the intervention group and 5 women for the control group, in each country)

   [Time Frame: Three to nine months]
6. Benefit of the intervention a future full-scale RCT trial
   Perception of the intervention by women participating in the pilot through the information obtained in qualitative interviews.

   [Time Frame: Three to nine months]
7. Perception about the delay of the intervention of the control group for a future full-scale RCT trial
   Perception about the duration of delay of the intervention of women in the control group (provided in the qualitative interviews).

[Time Frame: Three to nine months]

8. Reasons for acceptability, non-adherence, and obstacles for a future full-scale RCT trial
   Reasons for acceptability, non-adherence, and obstacles to recruitment, randomization, consent and follow-up (provided in the qualitative interviews).

   [Time Frame: Three to nine months]

9. Follow up rate for a future full-scale RCT trial
   Rate of failure to obtain data in the follow-up.

   [Time Frame: Three to nine months]

Eligibility

Minimum Age: 16 Years

Maximum Age:

Sex: Female

Gender Based: No

Accepts Healthy Volunteers: No

Criteria: Inclusion Criteria:

• Pregnant women at <12 weeks gestation, who screen positive in IPV at the first antenatal visit and accept the e-health package.

Exclusion Criteria:

- Women who cannot be informed about the study without their partners or other family members knowing
- Women mentally or physically incapacity to participate in the study
- Women below 16 years in Spain or below 18 years in Denmark
- Inability to understand Danish/Spanish
- Lack of internet and electronic device
- Women with extreme severity of IPV. Women selected to participate in the trial in this situation will receive a danger assessment before randomisation and if the severity of IPV is confirmed, they will be routinely treated and supported according to the standard protocol in each country. Women who have same-sex partners will be screened, but their data will not be used for the purpose of this study.

Contacts/Locations

Central Contact Person: Antonella Ludmila Zapata Calvente
Telephone: 958246270
Email: antonellalzc@ugr.es

Central Contact Backup: Aurora Bueno Cavanillas
Email: abueno@ugr.es

Study Officials: Khalid S. Khan
Study Director
Universidad de Granada

Locations: **Spain**
University of Granada
Granada, Spain
Contact: Antonella Ludmila Zapata Calvente
Principal Investigator: Antonella Ludmila Zapata Calvente, PhD

Principal Investigator: Stella Martín de las Heras, MD, PhD

Denmark
Odense University Hospital
Odense, Denmark
Contact: Vibeke Rasch
Principal Investigator: Vibeke Rasch, MD, PhD

IPDSharing

Plan to Share IPD: Yes
We will collaborate in an approved, registered Individual Participant Data metaanalysis

Supporting Information:
Study Protocol
Informed Consent Form (ICF)
Clinical Study Report (CSR)

Time Frame:
Starting 6 months after publication

Access Criteria:
On reasonable request, protocol, data collection forms and published results are available from investigators. Sharing would need to be comply with data protection laws.

URL:

References

Citations: Martin-de-Las-Heras S, Velasco C, Luna-Del-Castillo JD, Khan KS. Breastfeeding avoidance following psychological intimate partner violence during pregnancy: a cohort study and multivariate analysis. BJOG. 2019 May;126(6):778-783. doi: 10.1111/1471-0528.15592. Epub 2019 Jan 24. PubMed 30575266

Román-Gálvez RM, Martín-Peláez S, Martínez-Galiano JM, Khan KS, Bueno-Cavanillas A. Prevalence of Intimate Partner Violence in Pregnancy: An Umbrella Review. Int J Environ Res Public Health. 2021 Jan 15;18(2). pii: E707. doi: 10.3390/ijerph18020707. Review. PubMed 33467538

Rasch V, Van TN, Nguyen HTT, Manongi R, Mushi D, Meyrowitsch DW, Gammeltoft T, Wu CS. Intimate partner violence (IPV): The validity of an IPV screening instrument utilized among pregnant women in Tanzania and Vietnam. PLoS One. 2018 Feb 1;13(2):e0190856. doi: 10.1371/journal.pone.0190856. eCollection 2018. PubMed 29389954

Links:

Available IPD/Information: Type: Other [Any documents]
Contact authors for information
