# Supplementary material for: E-health psychological intervention in pregnant women exposed to intimate partner violence (eIPV): A protocol for a pilot randomised controlled trial
Source: PLoS One. 2023 Mar 17;18(3):e0282997. doi: 10.1371/journal.pone.0282997 (PMC10022801; doi:10.1371/journal.pone.0282997)
Supplement: S2 File — (PDF) [file pone.0282997.s004.pdf]

PROJECT MEMORY [MEMORIA DEL PROYECTO]

[TITLE: STop intimate partner violence in Pregnancy. Subproject: Pilot study of a randomized controlled clinical trial to evaluate the feasibility of a video-counseling support tool (e-Health) for pregnant women exposed to intimate partner violence.] **[TÍTULO:** STop intimate partner viOlence in Pregnancy (Parar violencia de género en el embarazo). Subproyecto: Estudio piloto de un ensayo clínico controlado aleatorio para evaluar la viabilidad de una herramienta de soporte por video-asesoramiento (e-Salud) para mujeres embarazadas expuestas a violencia por el compañero íntimo.]

PRINCIPAL INVESTIGATOR: Stella Martín de las Heras **[INVESTIGADOR PRINCIPAL:** Stella Martín de las Heras]

CENTER: FACULTY OF MEDICINE. UNIVERSITY OF GRANADA Department of Legal Medicine, Toxicology and Physical Anthropology **[CENTRO:** FACULTAD DE MEDICINA. UNIVERSIDAD DE GRANADA Departamento de Medicina Legal, Toxicología y Antropología Física]

FINANCING: European Program for Rights, Equality and Citizenship (REC-AG-2019), in the call for proposals to prevent and combat all forms of violence against children, youth and women (REC-RDAP-GBV-AG-2019). **[FINANCIACIÓN:** Programa Europeo de Derechos, Igualdad y Ciudadanía (REC-AG-2019), en la convocatoria de propuestas para prevenir y combatir todas las formas de violencia contra niños, jóvenes y mujeres (REC-RDAP-GBV-AG-2019)]

Abstract

Introduction. Intimate partner violence (IPV) during pregnancy, a condition as common as obstetrics conditions like gestational diabetes, is associated with maternal and neonatal complications. Systematic detection of IPV is not well established in antenatal screening probably because the effectiveness of protective interventions has not been evaluated. E-health interventions may be beneficial among mothers exposed to IPV. Prior to performing a full-scale effectiveness trial for such an intervention, a pilot

study is required to assess the feasibility of randomising a sufficiently large number of women exposed to IPV during pregnancy.

Methods. The eIPV trial is a randomised pilot study nested within a cohort of consenting mothers who screen positive for IPV in the first antenatal visit at <12 weeks' gestation and accept an e-health package (psychological counselling by videoconference) in Spain and Denmark. Twenty eligible mothers from the above cohort will be randomised to either intervention or control. The intervention group will receive the e-health package as part of the cohort. The control group will be invited to accept a delay in the intervention (e-health package eight weeks later). After consenting to delay, the control group will provide comparative data without losing the opportunity of obtaining the intervention. We will determine estimates of rates of informed consent to randomization, and the rates of adherence and dropout following randomization. Qualitative interviews will be conducted to examine the women's perception about the benefit of the intervention, reasons for acceptability and non-adherence, and obstacles to recruitment, randomisation and consent. The results will inform the trial feasibility and variance of key clinical outcome measures for estimation of sample size of the full-scale effectiveness trial.

Pilot study of a randomized controlled clinical trial to evaluate the feasibility of a video-counseling support tool (e-Health) for pregnant women exposed to intimate partner violence.

[***Estudio piloto de un ensayo clínico controlado aleatorio para evaluar la viabilidad de una herramienta de soporte por video-asesoramiento (e-Salud) para mujeres embarazadas expuestas a violencia por el compañero íntimo***]

Introduction

Background and rationale

Intimate partner violence (IPV) is one of the most common forms of violence against women and includes physical, sexual, and emotional abuse and controlling behaviour [1]. Globally, the lifetime prevalence of physical and sexual IPV for women is around 30%[2]. Violence during pregnancy is more common than preeclampsia or gestational diabetes which are routinely screened for antenatally[3]. In Spain, 3.6% of pregnant women have suffered physical IPV during pregnancy [4]. Although the true prevalence of IPV during pregnancy in EU is unclear, it is evident that a substantial minority of women experience violence during pregnancy[6]. It is estimated that 10% of hospitalizations are due to intentional injuries inflicted upon the pregnant woman[7]. Also, IPV during pregnancy has been linked to depression, both during pregnancy and in the postpartum period[8].

To improve the health of pregnant women and their infants, it is important that targeted IPV interventions are developed and implemented as part of routine antenatal care.

E-health tools are potential solutions to deliver effective treatment and support to women exposed to IPV. Trials have shown that e-health screening tools are effective in getting women to disclose or detect IPV[9–11]. Trials have also found that women who receive telephone counselling combined with other forms of support or a web-based safety decision aid kit are more likely to adopt safety behaviours than women in a control group, which were not offered e-health solutions[12–15]. It has been found that online safety planning is a feasible method among pregnant women[16].

Based on the available evidence, we have developed an e-health intervention package combining an online screening tool and video counselling sessions by trained providers for pregnant women who screen positive for IPV. Screening for IPV combined with an empowerment intervention that includes brief education and video counselling on safety

planning may have potential to address repeated IPV and associated adverse health effects among pregnant women. However, prior to performing a large interventional trial, a pilot study is needed to identify barriers to recruitment, assess feasibility and acceptability of the treatment, and fine-tune study procedures.

Objective

The objective is to assess the need and feasibility of randomising a sufficiently large number of women exposed to IPV during pregnancy in a full-scale future randomised trial. To achieve this, we will:

a) estimate rates of consent to randomization, and the rates of adherence and dropout following randomization (for the use in sample size estimation)
b) determine recruitment duration
c) examine the women's perception about the benefit of the intervention
d) determine the reasons for acceptability, non-adherence, and obstacles to recruitment, randomisation and consent through qualitative interviews

Trial design

A pilot randomised controlled trial (RCT), co-designed by patient input using Zelen's design (if permitted by the ethics committees) with additional qualitative evaluation, will be nested within a cohort study.

We will use a modified Zelen' design with a double consent process[17–19] and a delayed intervention for the control group. In the first stage, informed consent, for which ethics approval is already in place, will be sought from all pregnant women to enter a cohort study. A predetermined small number of the cohort will then be randomized, without their knowledge, to intervention or control (see figure 1). The intervention group will receive the e-health package as part of the cohort.

In the second stage, participants who have been assigned to the control, will be reapproached and given information about their participation in the control group. At this stage, they will be invited to accept a delay in the intervention (e-health package eight weeks later) and be asked to give the second informed consent for the delayed intervention. Those who decline, will remain in the cohort study. Those in the cohort only group, will not be informed about the randomization, as their subsequent follow up in the study will remain part of the cohort study to which they would already have

consented in the first stage. We will substitute the women who not consent being part of the control until we reach the sample required (5 women). This information will be also analysed for a future full-scale RCT.

Methods: participants, interventions and outcomes

Study setting

Participants will be recruited at twenty-nine urban public primary health antenatal care centers within Andalusia (Spain). Participants will be randomised to the intervention group or to the control group (figure 1).

Eligibility criteria

All women who fulfil the inclusion criteria will be screened and invited to receive an e-health package. Of those who accept the e-health intervention, we will randomise the first 10 women accepting during September 2021, 5 will be allocated in the intervention group and 5 will be asked to consent to be in the control group.

Inclusion criteria: pregnant women at <12 weeks gestation, who screen positive in IPV at the first antenatal visit and accept the e-health package.

Exclusion criteria: (1) women who cannot be informed about the study without their partners or other family members knowing; (2) mentally or physically incapacity to participate in the study; (3) women below 16; (4) inability to understand Spanish, and (5) lack of internet and/or an electronic device. Women who have same-sex partners will be screened, but their data will not be used for the purpose of this study. Women with an elevated risk for IPV will be routinely treated and supported according to the standard protocol in each country.

Recruitment: Consent procedures

In advance, a written, informed consent for administering the screening of IPV, will be sought from the pregnant women at the first antenatal visit with a midwife. Those who screen positive, will also be asked for consent to receive the e-health package intervention and to complete baseline and outcome measurement questionnaires (one month after the intervention). **The cohort study has already been granted ethics approval (study code: 881648; date of approval: 21/07/2020) as well as regarding the intervention and qualitative interviews (study code: 881648-2; date of approval: 28/01/2021).** For the pilot trial, women who consent to receive the e-health

package will be randomized (following a modified Zelen's design) into the intervention group (without their knowledge) or into the control group. Women in the control group will additionally be asked for a second informed consent, to be allocated in this group and to receive the e-health package 8 weeks later. This approach is suggested by the input of IPV survivors in a focus group already conducted. A few women from the intervention and control groups of the pilot trial will be asked for consent to be contacted for a qualitative interview. Women will be informed that they have the right to refuse participation as well as to withdraw their consent at any time, without giving a reason, and that this will not affect their subsequent care. If they withdraw consent, data collected up to the point of withdrawal will be retained in the study. Participant information sheets and consent forms used for this pilot study are available as supplementary files. The informed consent provides study aims, contact details and sources for further information. All recruitment materials have been developed with significant input from a patient and public involvement (PPI) (see the Discussion section).

Screening procedures

Eligible women are invited by trained midwives at the first antenatal care visit to fill in an app developed for tablets for the screening of IPV during pregnancy.

Women who screen positive in IPV will be invited to receive an e-health intervention (see the positivity criteria in the data collection section below).

Randomisation procedures

The research team will consult the data server on a daily basis to identify women who screen positive in IPV and accept to receive the e-health package to randomly allocate them (using a computer-generated random numbers) in the intervention or control group through randomisation at a ratio of 1:1. The IPV counsellor will be informed of the assignments to each group. Women in the intervention group will be blinded but not women in the control group.

Study interventions and assessments

Intervention group: Women positive for IPV who accept the e-Health intervention and who have been randomly allocated in the intervention group will receive the e-health package as the rest of the cohort, as well as the baseline and outcome measurements. The e-health package will include six video counselling sessions by trained provider - a

psychologist- and the access to a mobile application for designing security plans, an adapted version of the mobile application “My Plan”. We will monitor if participants adhere to all the sessions offered for counselling and in case of non-adherence or failure to engage in counselling, the counsellor will follow specific protocol to encourage reengagement. The psychologist will contact the woman once through the preferred mean (email or phone). The content of the six individually tailored sessions will be based on the Dutton’s Empowerment Model[20] and the Psychosocial Readiness Model[21]. Specifically, the contents will include the evaluation of abusive behaviour; safety planning, network and resources; psycho-education (healthy relationships, cycle of violence…); self-esteem and empowerment; fears; choice making and problem solving. Criteria for discontinuing or modifying allocated interventions will be applied to women with an elevated risk for IPV who will be routinely treated according to the standard protocol in each country.

Control group: women positive in IPV who accept the e-Health intervention package will be asked for a second consent to receive a delayed intervention (8 weeks later) and to complete the baseline and outcome measurements. Women can request to leave the control group at any time and to receive the intervention immediately (in which case they data will be part of the cohort study).

Assessments and data collection: data on socio-demographic characteristics and partner violence will be collected during the screening process. The validated screening questionnaires to detect IPV will be the short form of the Women Abuse Screening tool (WAST-Short)[22], a 2-items tool that measures conflict and tension and the Abuse Assessment Screen (AAS)[23], a 5-item screening questionnaire including emotional, physical, and sexual violence as well as fear of partner within the past year or ever. Data from previous studies support the reliability and validity of the WAST[24] and the AAS[23]. A woman is initially screened positive for exposure to IPV in the AAS if she answers “yes” to one or more of the five questions if the perpetrator is the partner or ex-partner . A woman screens positive in WAST-Short if she meets one of the two original criteria: having some or a lot of tension and/or some or a lot of difficulties to solve problems with her partner (all positive responses receive 1, the cut-off = 2) or if she indicates having a lot of tension and/or a lot of difficulties (extreme responses receive 1, cut-off =1). If the women initially are screened positive, the screening questionnaires will automatically be followed by the Index of Spouse Abuse (ISA) questionnaire[25]. The

ISA questionnaire is a detailed 30-item questionnaire about IPV (emotional, physical and sexual) in order to confirm the IPV and evaluate the severity of the violence. Two different scores are computed: ISA-P (physical abuse) and ISA-NP (non-physical abuse). The scores range from 0 to 100. Based on the Spanish validation of the ISA tool[26] that gives different weights to the original items, cut-off scores of 6 for ISA-P, and 14 for ISA-NP will be applied. The reliability (α) of the ISA range from .82[26] to .96[25].

After consent the e-health package, and to assess the impact of the intervention on a number of factors, quantitative questionnaire data will be collected before and after the e-health intervention. Exposure to IPV will be assessed by use of the ISA tool, and post-natal depression will be assessed by use of The Edinburgh Postnatal Depression Scale (EPDS, α = .81) which is a 10-item validated questionnaire designed to detect postnatal depression[27]. Further, participants will be asked to assess their ability to carry out safety behaviour actions by use of a revised version of the 22-item safety action checklist (α = .75)[28] and to complete a Measure of Victim Empowerment Related to Safety (MOVERS; α = .74-.88)[29] All the data will be entered into a bespoke secure online study database using a unique study ID for each participant. We will use the services of the online secure server Lime Survey hired by the University of Granada.

Qualitative data will be collected through individual in-depth interviews with a few women at any point of the flow diagram (Figure 1) to explore their opinion and experiences of the study procedures and intervention. Additionally, we will conduct interviews with psychologist and some midwives to explore their experiences with the delivery of the intervention and their opinion about a future full-randomised controlled trial. A semi-structured interview guide will be developed for the interviews using the Model for Assessment of Telemedicine Applications (MAST) as theoretical framework[30] with focus primarily on the following domains: users’ perspectives, their safety, ethical and organisational aspects; health problems and technology. All participants will give written consent prior to the interviews starting and data collection will stop once data is saturated. The interviews will be conducted in Spanish and audio recorded, and will be transcribed and analysed by use of a thematic content analysis using a combined inductive-deductive approach[31]. A coding frame will be developed, and themes will deductively be derived from MAST and supplemented with categories that inductively arise from the data. See Table 1 for an overview of intervention and

assessments.

Data will be collected by the psychologist and other researchers of the STOP Project. In case of failure to provide data in the follow-up assessments, the participant will be contacted by the preferred mean: phone or email. If a woman indicates she wants to be contacted by phone, the psychologist will make a maximum of three phone calls at different times and days. If the contact is made by email or phone message, the counsellor will send a maximum of two attempts leaving between both at least three days.

Relevant concomitant care and interventions that are permitted during the trial: Women in both the intervention and control groups would be able to benefit from the standard care available for women exposed to IPV thorough the usual provision made by social and health services at any time during the study, if necessary. Indeed, one of the video-counselling sessions will be dedicated to inform women about the available resources in their community. IPV counsellors will specify if women in the intervention and the control group are in need of these resources during the time of the pilot as a valuable information for the future full-scale trial.

Outcomes

To accomplish the objectives, we will collect the following information:

- Number of women who were positive in IPV and consent to receive e-health package
- Number of women who were positive in IPV, consent to receive e-health package and consent to randomization in the control group. Number of women who were approached and did not consent to be allocated in the control group.
- Number of women who were recruited to intervention group, and for whom complete outcomes were obtained.
- Number of women who were recruited to control group, and for whom complete outcomes were obtained.
- Recruitment duration to get the pilot sample (5 women for the intervention group and 5 women for the control group, in each country)
- Benefit of the intervention perceived by women.

- Reasons for acceptability, non-adherence, and obstacles to recruitment, randomisation and consent (through qualitative interviews).
- Rate of failure to obtain data in the follow-up.

Participant timeline

The participant timeline is illustrated in Table 1.

Table 1. Schedule of enrolment, interventions and assessments

	Study period					
Time-point	**Enrollment**	**Screening**	**Allocation**	**Post-allocation**		**Follow-up**
				Baseline measurement	Outcome measurement	
ENROLLMENT						
Allocation (randomisation)			X			
Eligibility screen	X	X				
Informed consent		X	X			
INTERVENTION						
Cohort			X			
Intervention			X			
Control			X			
ASSESSMENTS						
Demographic information		X				
WAST-Short		X				
AAS		X				
ISA		X			X	
EPDS				X	X	
Safety action checklist				X	X	
MOVERS				X	X	
Qualitative interviews						X

Statistics

Sample size calculation

The pilot study will not be powered to detect statistical differences in key clinical outcomes, but the sample sizes have been chosen to highlight problems and confirm the potential to detect differences. We will review patient and staff feedback before finalising a protocol for a full-scale multicentre RCT suitable for an application for EU research funding.

Statistical analysis

To determine the feasibility, we will perform the analysis against established stop-go rules for reporting to comply with the CONSORT statement for pilot and feasibility trials [32]. The feasibility analysis will focus on the outcomes described above. We will also collect information within the cohort study (see Discussion section).

Progression criteria

Table 2 below outlines the main criteria that will be considered to assess the feasibility of a full-scale RCT. In addition, our qualitative findings will also be used to support the decision-making around progression to a full-scale trial. For example, if a progression criterion outlined in table 2 does not meet the threshold for progression, but we have developed a qualitative understanding of why this occurred and how it could be improved, then it may still be possible to proceed with the full trial. The research team of the STOP project will have access to these interim results.

Table 2 Suggested progression criteria

**Feasibility objectives and related data to be collected**	**Go criteria to proceed to full trial**	**Criteria to reassess and adjust full trial protocol**	**Stop criteria**
Study population			
1. Consent rate of eligible women	Rate >25% of eligible women agreeing to participate.	Rate between 11% and 24% women agreeing to participate	Rate <10% of eligible women agreeing to participate
Study outcomes			
2. Proportion of women in either intervention or control group for whom the allocated treatment is adhered to.	Adherence to allocated treatment in >80% of study sample.	Adherence to allocated treatment in between 51% and 79% of study sample.	Adherence to allocated treatment in <50% of study sample.
RCT process			
3. Collection of data on clinical outcomes	Complete data available of >80% of study sample.	Missing data between 21% and 49% of study sample.	Data missing of >50% of study sample.

Trial management and monitoring

Data management

All data management will be undertaken by the University of Granada (UGR). Standard operating procedures will be in place for the collection and handling of data. All study data will be entered directly by trained and delegated research staff included in the STOP project into a secure, bespoke electronic trial database with inbuilt range checks set up and hosted by the University of Granada. User accounts will be allocated and managed centrally by the trial coordinator and restricted to appropriate site level access. Data collected on the forms and entered into the electronic database will only identify

the participants by a unique trial number. No identifiable data will be stored in the trial database.

Trial management

The trial is managed and run by the University of Granada. This center is responsible for safety reporting, coordination of trial committees, statistical analysis and reporting, trial monitoring, database management and case report form design.

Pilot trial oversight

The Advisory board has been established to oversee and monitor the trial conduct and patient safety. The board is chaired by an independent professor from Oslo (Dr Mirjam Lukasse, University of South-Eastern Norway), with two other independent members, professor experts in IPV, from Copenhagen (Denmark; Prof Tine Gammeltoft) and Alicante (Spain; Dr Carmen Vives Cases). The PSC provides overall supervision of the trial and ensures that it is being conducted according to the protocol, good clinical practice and relevant regulations. This committee also monitors trial progress in relation to recruitment, data capture and completeness, protocol adherence and deviations and subject withdrawals. The committee will meet at the request of the investigators. The PSC will be also responsible for reviewing the trial data throughout the study and assessing whether there are any safety issues that need to be brought to the attention of the sponsor, or any ethical reasons why the trial should not continue. Given the low risk of the study intervention and that it is non-blinded, no separate data safety monitoring committee will be established. The sponsor retains the right to audit the study, including any study site or central facility.

Safety reporting

The detection of severe or life-threatening, abuse of the pregnant woman by the IPV counsellor will be reported to the principal investigator of the country and these women will we treated according to the standard protocol in each country. The study should not add any risk or harm for the women, for this reason, we will not provide a post-trial care for compensation. On the contrary, it is anticipated that video counselling addressing safety behavior and safety planning has the potential to increase safe behaviors and thus decrease IPV exposure. We also anticipate that the participating midwives will experience a greater competence and confidence in approaching the topic of violence and handling women who are exposed to IPV. Women will benefit from health

professionals who enquire in an appropriate way about violence. In addition, during the video counselling sessions the IPV counselors will inform women of other community women's resources to use them if women need them when the e-health intervention is finished.

Patient and public involvement

A woman previously exposed to IPV will be the target representative. She will help to develop a more pregnant women-centred information sheet. The qualitative research embedded within this pilot study will prove integral in evaluating how the consent materials and processes were received in practice.

DISCUSSION

Even though numerous intervention models to address IPV have been developed, current efforts suffer from limitations. First, IPV services are often not integrated within routine health service delivery, and second, models for integrating IPV service in health service delivery where developed tend not to have generalizability to the European context[9–15]. To address the personally and politically sensitive problems associated with IPV among pregnant women, effective, sustainable, and culturally appropriate health system-based interventions need development. If found effective, e-health interventions would be suitable for incorporation in these care pathways.

Screening for IPV combined with an empowerment intervention that includes education and video counselling on safety planning may have potential to address repeated IPV and associated adverse health effect among pregnant women. The option of receiving counselling and support through video consultation during antenatal care presents an opportunity for more accessible and flexible care addressing some of the barriers associated with in-person care, such as travel distance and time, travel costs, and the stigma of seeking help. In addition, patients may be more motivated to seek and continue treatment if they are in a familiar environment of their own choice and can avoid stressful situations, such as navigating a hospital or maternity facility. The development of the video counselling intervention will capture the need for safety and adhere to strict security features preventing the risk of women's partners prying on their online activity. These provisos need underpinning effectiveness evidence.

Prior to performing a large interventional trial, a pilot study is needed to identify barriers to recruitment, assess feasibility and acceptability of the treatment, and fine-

tune study procedures. No RCT has previously assessed an e-health intervention in IPV among pregnant women in comparison with a control group (with a delay intervention). In this pilot trial, we chose to perform a pilot RCT with a modified Zelen's design rather than a pilot RCT for several reasons. Participants who take part in standard RCTs will make a judgment of their preferred treatment and often expect to be allocated to the treatment group[33]. If this does not occur, it can be followed by dissatisfaction and distrust in those who approached them to take part[34]. Consequently, randomization to a control group may lead to dropout after allocation. The original Zelen design involved randomization before consent, with consent only required from those allocated to the intervention, whereas the control group receive their usual care[19]. Baseline outcomes are collected from medical records (with ethical approval). However, it is not possible to have interaction with the control group during follow-up, as they are not informed of their presence in a study. Taking all of this into consideration, we hypothesized that women will accept the intervention when they perceive it as a need for support but it is also expected that if they perceive it like this, they may not want to be randomized into control group. To overcome this expected drop out in the control group, we follow the input of IPV survivors in a focus group, the opinion of the participant representative and a systematic review that concluded that a delay intervention could be an effective way of minimizing dropout[35]. In our pilot, thus we chose to offer to women of the control group a delay in the intervention.

Beyond the information described to be collected during this pilot, we will capture other relevant information in the cohort study useful for planning the future full-scale randomised control trial: number of women who were approached and agreed to fill in the IPV screening question, number of women where there was a study protocol violation, number of women exposed to physical IPV captured by the screening questions, number of pregnant women exposed to physical IPV that accept to participate in the intervention and in the control group, number of women willing to participate in follow-up interviews, the acceptability of the e-health package for ongoing IPV prevention and the acceptability of video-counselling for IPV prevention in terms of compliance to the scheduled counselling sessions.

In conclusion, the pilot study nested within the cohort study will allow us to obtain information about the rates of IPV in pregnancy, the acceptability of an e-health intervention and the availability of participants for randomisation into an effectiveness

trial. These results will inform us about the feasibility and variance of key clinical outcome measures for estimation sample size of the full-scale effectiveness trial.

Trial status

Protocol version 1.0, 30ht April 2021.

ETHICS AND DISSEMINATION

The trial is applying for ethics approval to the Andalusian Research Ethics Committee. All subjects participating in the trial will provide written informed consent where possible as highlighted in the methods section above. Specific exceptions to this have been approved by the Confidentiality Advisory Group. Any changes to the protocol are subject to a formal amendment and may not be implemented prior to the approval by the Research Ethics Committee.

Confidentiality

In order to protect confidentiality before, during, and after the trial, personal information about potential and enrolled participants will be collected and saved during the screening process in a secured data base and it will be only accessed by authorised research investigators of the STOP project. The information of the baseline and outcome measurements will be saved in different databases, and both could be only aggregated by the research members by the birthdate and telephone number of the women.

Declarations of interests

Financial and other competing interests for principal investigators for the overall trial and each study site.

Access to data

Research team members from the STOP project will have access to the final pilot trial dataset only for research purposes according to the objectives established in this protocol.

Dissemination policy

We will share the eHealth package with relevant stakeholders in order to upscale it across the EU as well as globally to better combat IPV. Concrete dissemination activities include the production of research papers, guidelines and white papers

detailing the eHealth intervention, which will be made available on the STOP project website and presented at relevant professional conferences reaching both health care professionals and other relevant stakeholders and policymakers.

Consent for publication

All relevant data from this study will be submitted to peer-reviewed journals for publication following the completion of the study in line with sponsor publication policy. Data will be captured for all study participants, and no patient identifiable data will be used in any publications. The sponsor retains the right to review all publications prior to submission or publication. Responsibility for ensuring accuracy of any publication from this study is delegated to the chief investigator. Authorship will be assigned in compliance with International Committee of Medical Journal Editors (ICMJE) guidelines.

REFERENCES

1. Organization WH, others. Understanding and addressing violence against women: intimate partner violence. Published online 2012. Accessed June 18, 2016. http://apps.who.int/iris/handle/10665/77432

2. García-Moreno C, Pallitto C, Devries K, Stöckl H, Watts C, Abrahams N. *Global and Regional Estimates of Violence against Women: Prevalence and Health Effects of Intimate Partner Violence and Non-Partner Sexual Violence*. World Health Organization; 2013.

3. Parsons L, Goodwin MM, Petersen R. Violence Against Women and Reproductive Health: Toward Defining a Role for Reproductive Health Care Services. *Matern Child Health J*. 2000;4(2):6.

4. Martin‐de‐las‐Heras S, Velasco C, Luna‐del‐Castillo JD, Khan KS. Breastfeeding avoidance following psychological intimate partner violence during pregnancy: a cohort study and multivariate analysis. *BJOG Int J Obstet Gynaecol*. 2019;0(0). doi:10.1111/1471-0528.15592

5. Lukasse M, Schroll A-M, Karro H, et al. Prevalence of experienced abuse in healthcare and associated obstetric characteristics in six European countries. *Acta Obstet Gynecol Scand*. 2015;94(5):508-517. doi:10.1111/aogs.12593

6. Lukasse M, Schroll A-M, Ryding EL, et al. Prevalence of emotional, physical

and sexual abuse among pregnant women in six European countries. *Acta Obstet Gynecol Scand*. 2014;93(7):669-677. doi:10.1111/aogs.12392

7. Chambliss LR. Intimate partner violence and its implication for pregnancy. *Clin Obstet Gynecol*. 2008;51(2):385-397. doi:10.1097/GRF.0b013e31816f29ce

8. Martin SL, Li Y, Casanueva C, Harris-Britt A, Kupper LL, Cloutier S. Intimate Partner Violence and Women's Depression Before and During Pregnancy. *Violence Women*. 2006;12(3):221-239. doi:10.1177/1077801205285106

9. Ahmad F, Hogg-Johnson S, Stewart DE, Skinner HA, Glazier RH, Levinson W. Computer-Assisted Screening for Intimate Partner Violence and Control. *Ann Intern Med*. 2009;151(2):93-102. doi:10.7326/0003-4819-151-2-200907210-00124

10. Klevens J, Sadowski L, Kee R, Trick W, Garcia D. Comparison of Screening and Referral Strategies for Exposure to Partner Violence. *Womens Health Issues*. 2012;22(1):e45-e52. doi:10.1016/j.whi.2011.06.008

11. Rhodes KV, Drum M, Anliker E, Frankel RM, Howes DS, Levinson W. Lowering the threshold for discussions of domestic violence: a randomized controlled trial of computer screening. *Arch Intern Med*. 2006;166(10):1107-1114. doi:10.1001/archinte.166.10.1107

12. McFarlane J, Malecha A, Gist J, et al. An intervention to increase safety behaviors of abused women: results of a randomized clinical trial. *Nurs Res*. 2002;51(6):347-354. doi:10.1097/00006199-200211000-00002

13. Gillum TL, Sun CJ, Woods AB. Can a Health Clinic-Based Intervention Increase Safety in Abused Women? Results from a Pilot Study. *J Womens Health*. 2009;18(8):1259-1264. doi:10.1089/jwh.2008.1099

14. Koziol-McLain J, Vandal AC, Wilson D, et al. Efficacy of a Web-Based Safety Decision Aid for Women Experiencing Intimate Partner Violence: Randomized Controlled Trial. *J Med Internet Res*. 2018;19(12):e426. doi:10.2196/jmir.8617

15. Tiwari A, Yuk H, Pang P, et al. Telephone intervention to improve the mental health of community-dwelling women abused by their intimate partners: a randomised controlled trial. *Hong Kong Med J Xianggang Yi Xue Za Zhi*. 2012;18 Suppl 6:14-17.

16. Bloom TL, Glass NE, Case J, Wright C, Nolte K, Parsons L. Feasibility of an Online Safety Planning Intervention for Rural and Urban Pregnant Abused Women.

Nurs Res. 2014;63(4):243-251. doi:10.1097/NNR.0000000000000036

17. Land J, McCourt O, Heinrich M, et al. The adapted Zelen was a feasible design to trial exercise in myeloma survivors. *J Clin Epidemiol*. 2020;125:76-83. doi:10.1016/j.jclinepi.2020.04.004

18. Schellings R, Kessels AG, ter Riet G, Knottnerus JA, Sturmans F. Randomized consent designs in randomized controlled trials: Systematic literature search. *Contemp Clin Trials*. 2006;27(4):320-332. doi:10.1016/j.cct.2005.11.009

19. Zelen M. A New Design for Randomized Clinical Trials. *N Engl J Med*. 1979;300(22):1242-1245. doi:10.1056/NEJM197905313002203

20. Dutton MA. *Empowering and Healing the Battered Woman: A Model for Assessment and Intervention*. Springer Publishing Co; 1992:xx, 202.

21. Cluss PA, Chang JC, Hawker L, et al. The process of change for victims of intimate partner violence: support for a psychosocial readiness model. *Womens Health Issues Off Publ Jacobs Inst Womens Health*. 2006;16(5):262-274. doi:10.1016/j.whi.2006.06.006

22. Brown JB, Lent B, Schmidt G, Sas G. Application of the Woman Abuse Screening Tool (WAST) and WAST-short in the family practice setting. *J Fam Pract*. 2000;49(10):896-903.

23. Soeken KL, McFarlane J, Parker B, Lominack MC. The Abuse Assessment Screen: A clinical instrument to measure frequency, severity, and perpetrator of abuse against women. In: *Empowering Survivors of Abuse: Health Care for Battered Women and Their Children*. Sage series on violence against women. Sage Publications, Inc; 1998:195-203.

24. Rabin RF, Jennings JM, Campbell JC, Bair-Merritt MH. Intimate Partner Violence Screening Tools. *Am J Prev Med*. 2009;36(5):439-445.e4. doi:10.1016/j.amepre.2009.01.024

25. Hudson WW, McIntosh SR. The Assessment of Spouse Abuse: Two Quantifiable Dimensions. *J Marriage Fam*. 1981;43(4):873. doi:10.2307/351344

26. Ruiz Pérez, Isabel, Plazaola Castaño, Juncal, Escribá Agüir, Vicenta, Jiménez Martín, Juan Manuel. *Adaptación Española de Un Instrumento de Diagnóstico y Otro de Cribado Para Detectar La Violencia Contra La Mujer En La Pareja Desde El*

Ámbito Sanitario. Observatorio de Salud de la Mujer. Escuela Andaluza de Salud Pública; 2006:56.

27. Malin Eberhard-Gran SO Anne Eskild, Kristian Tambs, Berit Schei. The Edinburgh Postnatal Depression Scale: Validation in a Norwegian community sample. *Nord J Psychiatry*. 2001;55(2):113-117. doi:10.1080/08039480117684

28. Ford-Gilboe M, Varcoe C, Scott-Storey K, et al. Longitudinal impacts of an online safety and health intervention for women experiencing intimate partner violence: randomized controlled trial. *BMC Public Health*. 2020;20(1):260. doi:10.1186/s12889-020-8152-8

29. Goodman LA, Cattaneo LB, Thomas K, Woulfe J, Chong SK, Smyth KF. Advancing domestic violence program evaluation: Development and validation of the Measure of Victim Empowerment Related to Safety (MOVERS). *Psychol Violence*. 2015;5(4):355-366. doi:10.1037/a0038318

30. Kidholm K, Ekeland AG, Jensen LK, et al. A model for assessment of telemedicine applications: mast. *Int J Technol Assess Health Care*. 2012;28(1):44-51. doi:10.1017/S0266462311000638

31. Schreier, Margrit. *Qualitative Content Analysis in Practice*. London. Sage; 2013.

32. Eldridge SM, Chan CL, Campbell MJ, et al. CONSORT 2010 statement: extension to randomised pilot and feasibility trials. *Pilot Feasibility Stud*. 2016;2:64. doi:10.1186/s40814-016-0105-8

33. King M, Nazareth I, Lampe F, et al. Impact of Participant and Physician Intervention Preferences on Randomized Trials: A Systematic Review. *JAMA*. 2005;293(9):1089. doi:10.1001/jama.293.9.1089

34. Featherstone K, Donovan JL. "Why don't they just tell me straight, why allocate it?" The struggle to make sense of participating in a randomised controlled trial. *Soc Sci Med*. 2002;55(5):709-719. doi:10.1016/S0277-9536(01)00197-6

35. Bisschop CNS, Courneya KS, Velthuis MJ, et al. Control Group Design, Contamination and Drop-Out in Exercise Oncology Trials: A Systematic Review. *PLOS ONE*. 2015;10(3):e0120996. doi:10.1371/journal.pone.0120996

MEMBERS OF THE INVESTIGATION TEAM

[MIEMBROS DEL EQUIPO INVESTIGADOR]

- Principal Investigator [Investigador principal]: Stella Martín de las Heras (F): Catedrática MD, PhD.
- Senior Investigator [Investigador senior]: Aurora Bueno: Catedrática, MD, PhD. Responsible for the intervention. [Responsable de la intervención.]
- Senior Investigator [Investigador senior]: Khalid Saeed Khan: Ginecólogo, MBBS, MSC. Responsible for the feasibility of the study. [Responsable de la viabilidad del estudio.]
- Senior Investigator [Investigador senior]: Jesús López Megías (M): Professor in psychology. Special advisor in psychology. [Catedrático en psicología. Asesor especial en psicología]
- Postdoctoral Investigator [Investigadora post-doc]: Antonella Ludmila Zapata Calvente. Doctor in psychology in the area of intimate partner violence against women. [Doctora en psicología en el área de violencia de pareja contra las mujeres]
- Psychologist [Psicóloga que realiza la intervención e-salud]: Sabina de León de León. Experience in counselling for victims of violence by intimate partners. [Experiencia en la atención a víctimas de violencia por parte del compañero íntimo.]
- Training advisor [Asesor de formación]: Juan Miguel Martínez Galiano: matrona. Responsible for the training of midwives. [Responsable de la formación de las matronas]
- Technical expert [Experto técnico]: Juan Carlos Torres Cantero: Profesor, PhysD, PhD. Responsible for the development of the screening tool. [Responsable del desarrollo de la herramienta de cribado]
